# tranSMART

**DOI:** 10.1186/2043-9113-5-S1-S9

**Published:** 2015-05-22

**Authors:** Mariska Bierkens, Wim van der Linden, Kees van Bochove, Ward Weistra, Remond J A Fijneman, Rita Azevedo, Jan-Willem Boiten, Jeroen Beliën, Gerrit A Meijer

**Affiliations:** 1 Tumor Profiling Unit – VUmc Cancer Center, Amsterdam, the Netherlands; 2 Philips Research, Eindhoven, the Netherlands; 3 The Hyve, Utrecht, the Netherlands; 4 eScience Center, Amsterdam, the Netherlands; 5 Center for Translational Molecular Medicine, Eindhoven, the Netherlands

## Characterisation

Open source data integration platform for translational research.

## Description

Translational researchers investigate how molecular alterations correlate to specific disease phenotypes and whether these alterations could be used in a diagnostic or therapeutic manner. In order to allow researchers to (re)examine previously generated molecular data, a solution for sustainable management of this data in an accessible manner is crucial. In the Netherlands, the Translational Research IT (TraIT) project initiated by the Center for Translational Molecular Medicine (CTMM) aims to provide end-to-end solutions for sustainable management and analysis of data by establishing a long-lasting IT infrastructure. The tranSMART platform is a cornerstone of its infrastructure, in which processed ‘final’ data of various domains become available for data-integration, querying, visualisation and analysis.

tranSMART [1] was developed by Johnson&Johnson and initially served as an internal research data warehouse. Made open source in 2012, projects and institutions such as IMI eTRIKS, Janssen, Pfizer, Sanofi, the tranSMART Foundation and CTMM-TraIT have since contributed to its further development [2]. Connections between tranSMART and tools such as OpenClinica (clinical information), XNAT (imaging), Molgenis Catalogue (biobanking), Galaxy (processing pipelines, experimental data) and Phenotype Database (experimental data) are being established. Per domain, users will be able to turn raw data obtained from patient samples into processed data for export into tranSMART, while from tranSMART it will be possible to trace back the original raw data and processing pipelines.

**Figure 1:**
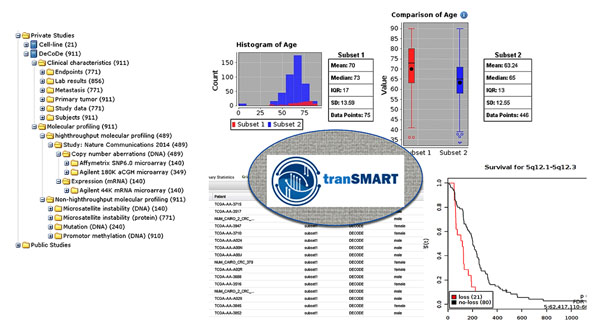
TransMART user interface. Display of data in tree style overview, summary statistics, gridview and advanced workflow analysis (aCGH survival).

Via tranSMART users may explore data (Figure 1) at various levels without needing to become domain experts to interpret the data. Data-type experts however will be capable of examining data in more detail using tools they are familiar with, or ones provided by CTMM-TraIT. In summary, tranSMART creates a window to complex bioinformatics data sets for disease experts with only limited understanding of specific bioinformatics pipelines.

## Status of development

tranSMART1.2 release in deployment “start of 2015”.

## Users

CTMM-TraIT, IMI eTRIKS, Janssen, Pfizer, Sanofi, the tranSMART Foundation, and many others.

## Link

http://transmartfoundation.org/
